# Purines in Pain as a Gliopathy

**DOI:** 10.3389/fphar.2021.649807

**Published:** 2021-03-10

**Authors:** Giulia Magni, Stefania Ceruti

**Affiliations:** Department of Pharmacological and Biomolecular Sciences, Università degli Studi di Milano, Milan, Italy

**Keywords:** microglia, astrocytes, satellite glial cells, A3 adenosine receptors, P2X4 receptors, P2Y12 receptors

## Introduction: Why is Pain Currently Considered as a “Gliopathy”

In the last decades, the hypothesis of the role of purinergic receptors expressed by glial cells in the regulation of pain pathways has become a matter of fact. Although several painful conditions are undoubtedly of neuronal origin, the existence of an altered bidirectional signaling between neurons and activated glial cells has brought to the definition of pain as a “gliopathy” (Ji et al., 2013). In the central nervous system (CNS), neuronal excitability in pain pathways is enhanced by resident microglia, astrocytes, oligodendrocytes, and by infiltrating cells, such as T cells and macrophages, overall constituting the so-called “neuroimmune interface.” Following peripheral nerve damage, spinal cord microglia sense a variety of neuron-derived stimuli leading to their transition toward a reactive state (Grace et al., 2014), whose inhibition through the antibiotic minocycline has proven anti-hyperalgesic and anti-allodynic (Wieseler et al., 2017). Microglia activation is followed by immune cell recruitment and by the appearance of a neurotoxic astrocyte subpopulation, the so-called A1 astrocytes (Liddelow et al., 2017). The extension of microglial and astrocytic processes into and near the synaptic space induces alterations to thousands of synapses, resulting in altered neural networks, maladaptive synaptic reorganization and generating a pro-inflammatory milieu, whose effects can extend for months after the initial nerve damage and guide the transition from acute to chronic pain (Gwak et al., 2017; Skaper et al., 2018). Neuronal firing is also significantly modulated at the sensory ganglion level, where neuronal bodies are surrounded and wrapped by resident satellite glial cells (SCGs), which become activated upon inflammation or nerve damage (Donegan et al., 2013; Magni et al., 2015), release neurotransmitters and chemical mediators and contribute to neuronal sensitization (Spray & Hanani, 2019). On the other way round, both CNS and PNS sensitized neurons increase their firing and release of neurotransmitters and neuromodulators that act paracrinally on glial cells, giving rise to a vicious and autoamplifying circle. Glial cells become permanently activated as demonstrated both in animal models of chronic pain and in patients (Loggia et al., 2015).

In order to identify new potentially “druggable” targets, research is now focusing on the identification of the whole network of molecules involved in neuron-to-glia communication in pain. In this scenario, multiple adenosine and nucleotide receptors are expressed by glial cells involved in pain transmission (for review see Burnstock, 2017; Magni et al., 2018; Magni & Ceruti, 2019), and their pharmacological modulation could represent an innovative analgesic strategy. In the next sections we shall contribute with our point of view on the role of P2 nucleotide and P1 adenosine receptors expressed by glial cells in modulating pain transmission, with the aim of identifying new and more effective approaches to painful conditions with respect to currently available drugs, which mostly target neurons.

## Purinergic Agents Targeting Glial Cells to Treat Pain: What is Currently Known

### Nucleotide Receptors as Key Modulators of Glia Reaction to Injury and Inflammation

Microglial cells are physiological guardians and modulators of neuronal activity, which can protect brain tissue from excessive neuronal firing. This fundamental function is mostly achieved by the ability of microglia to sense ADP, generated by the rapid breakdown of neuronal ATP released as cotransmitter, through membrane P2Y_12_ receptors which in turn control microglia chemotaxis and process dynamics (Badimon et al., 2020) and triggers a cascade of inhibitory events on neurons (Merlini et al., 2021). However, under chronic inflammatory conditions, in the presence of nerve injury or in chronic migraine, microglia become overactivated and shift toward a detrimental state, which involves overexpressed P2Y_12_ receptors (Jing et al., 2019; Yu et al., 2019). Thus, P2Y_12_ selective antagonists (i.e., thienopyridines cangrelor and prasugrel and the more recent nonthienopyridine Ticagrelor), which have been utilized for decades as antiplatelet agents, represent the starting point for the development of a microglia-mediated approach to chronic pain. Actually, a reduction of migraine headache symptoms was observed in patients with patent foramen ovale receiving thienopyridines as antiplatelet therapy (Sommer et al., 2018). These data would need additional confirmations to be exploited in migraine of different origin. Interestingly, P2Y_12_ receptors are only expressed by SGCs in sensory ganglia upon nerve injury and their inhibition reduced mechanical and heat hypersensitivity (Sugawara et al., 2017), thus confirming P2Y_12_-mediated pro-algogenic actions in various painful conditions. Other members of the G protein-coupled P2Y receptor family expressed by SGCs are recruited in chronic pain, including the P2Y_14_ (Lin et al., 2019) and the P2Y_2_ subtypes (Magni et al., 2015) and likely represent additional interesting targets.

The pivotal role played by ionotropic P2X4 nucleotide receptors in promoting neuronal sensitization after nerve injury has been firmly established over the past 20 years. As mentioned above, nerve injury triggers a complex signaling pathway leading to receptor upregulation in microglia, which in turn promotes the release of BDNF sensitizing second order neurons and causing the development of allodynia, as extensively reviewed elsewhere (Inoue, 2019). It is worth mentioning that the involvement of microglial P2X4 receptors in pain is sexually dimorphic, occurring in males and not in females (Tam & Salter, 2020), thus adding further complexity to an already complex scenario and suggesting the issue of sex must be taken into serious consideration in clinical trials.

Upregulation of brainstem microglial P2X4 receptors, which in turn promotes the release of pro-algogenic CGRP, has been also observed in a mouse model of migraine triggered by the recurrent administration of nitroglycerin (Long et al., 2020). Interestingly, novel discoveries have demonstrated a dual neuronal and microglial localization for P2X4 receptors (with controversial data in astrocytes and oligodendrocytes), which are mainly found intracellularly under physiological conditions but whose membrane expression is fostered by pathological stimuli (Duveau et al., 2020). Although the lack of really selective pharmacological tools has sometimes slowed down the discrimination between neuronal and glial P2X4-mediated effects, increased nucleotide concentrations and receptor expression make it possible to foresee a targeted action of P2X4 ligands at sites of pathological neuron-glia crosstalk.

Finally, it has been known for years that nucleotide receptors orchestrate reactive astrogliosis (for review see: Franke & Illes, 2014), but their role in chronic pain has not been clarified yet.

### The Emerging Role for Adenosine in Pain Modulation

Despite adenosine has been long considered as a simple neuromodulator with the main role to counterbalance and fine-tune nucleotide-mediated actions (Chen et al., 2013), this view has been progressively integrated with the demonstration that adenosine receptors, widely expressed throughout the body, play fundamental roles in many physiological and pathological conditions (Borea et al., 2018). This is particularly true for pathologies in which the local extracellular concentrations of nucleotides (and of their breakdown products nucleosides) rise several folds, such as tissue damage and hypoxia, but also during increased neuronal firing as in epilepsy and chronic pain. Many clinical trials involving adenosine receptor ligands have been carried out so far in various pathological conditions, but only a few compounds have been approved since 1990, i.e., the A_2A_ receptor agonist Regadenoson as pharmacological stress agent for myocardial perfusion imaging (MPI) and the selective A_2A_ receptor antagonist Istradefylline as an add-on to levodopa/carbidopa treatment for Parkinson’s disease (Jacobson et al., 2019; Chen & Cunha, 2020).

Concerning pain, adenosine receptors are expressed by glial cells and participate to their communication with neurons both in the CNS and in peripheral ganglia (Agostinho et al., 2020). Therefore, these receptors undoubtedly represent a promising approach to manage pain conditions characterized by a dysregulated cross-talk among different cell types. Available and sometimes contradictory data on the antinociceptive effects exerted by the administration of adenosine ligands in several preclinical models of acute and chronic pain have been recently extensively reviewed (Vincenzi et al., 2020). We shall now briefly highlight the most promising hints for their possible exploitation in humans as well.

The very first hypothesis of a contribution of adenosine receptors in pain was based on the role of the A_1_ and A_2A_ subtypes but, despite their efficacy in preclinical studies, selective agonists could not be included in clinical trials due to significant cardiovascular side effects (Jacobson et al., 2020). Nowadays, the most promising compounds under development are A_3_ receptor agonists: preclinical data showed that they are able to revert signaling dysregulation in cancer, autoimmune disorders and different pain conditions (Doyle et al., 2020). When compared to A_1_ and A_2A_ receptors, A_3_ receptors have low CNS expression levels, but are highly expressed in human immune and glial cells, including astrocytes, oligodendrocytes, microglia/macrophages and endothelial cells (Zhang et al., 2016), i.e., cell populations that are mostly involved in chronic painful conditions. In the last five years, the A_3_ receptor subtype has progressively emerged as innovative target for the control of pain, also thanks to the availability of selective pharmacological modulators, starting from IB-MECA and Cl-IB-MECA up to new more selective and potent agonists, including MRS5698 and MRS5980, which proved their efficacy in preclinical models of pain, including formalin-induced inflammatory pain and diabetic neuropathy (Magni et al., 2018; Jacobson et al., 2019; 2020).

Noteworthy, it has been demonstrated that dysregulation of A_3_ adenosine receptors recruitment is at the basis of chemotherapy-induced pain (Wahlman et al., 2018), but A_3_ receptor agonists do not interfere with antitumor effects of widely used chemotherapeutics; rather, they act as antitumor agents themselves. In fact, Cl-IB-MECA is currently in Phase II clinical trials for hepatocellular carcinoma as anticancer agent, and evidence suggest that activation of A_3_ receptors could provide dual benefits in the treatment of a variety of cancer-related pain states (Jacobson et al., 2020). As for chronic pain states, A_3_ agonists can be used as a monotherapy or in combination with opioids to improve their safety and efficacy against chronic pain, without reducing opioid’s antinociceptive effects (Doyle et al., 2020). Taken together, available data strongly support the further development of selective A_3_ receptor agonists for the treatment of different types of pain syndromes.

## Conclusion

**FIGURE 1:**
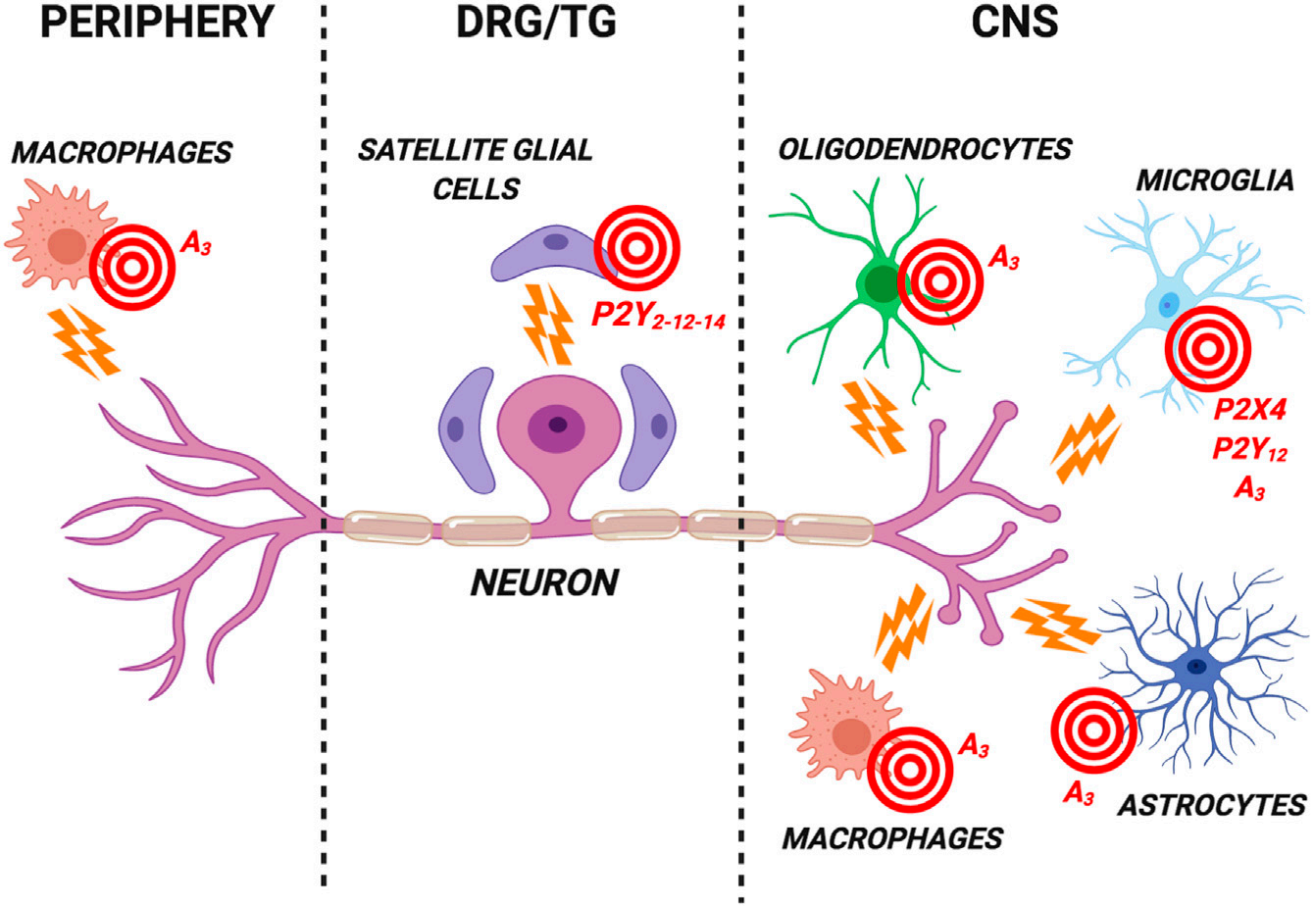
Schematic representation of the most promising purinergic targets for the development of new analgesics expressed by glial cells in the central nervous system (CNS), in dorsal root ganglia/trigeminal ganglia (DRG/TG) and by peripheral cells. These nucleotide (i.e., the P2Y_12_ and P2X4 subtypes) and adenosine (i.e., the A_3_ subtype) receptors are involved in the modulation of neuronal firing in chronic pain conditions. Receptor antagonists are likely to represent the best analgesic option targeting P2Y_12_ and P2X4 subtypes. Conversely, selective A_3_ receptor agonists have proved effective in reducing pain in several preclinical models. See text for details. Created with BioRender.com.

As for most of the discoveries on the purinergic system, the hypothesis of a purinergic modulation of pain plunges its roots in the intuitions of Geoffrey Burnstock who highlighted the P2X3 neuronal subtype as one of the fundamental receptors controlling visceral, inflammatory and neuropathic pain (Jarvis, 2020). However, several P2X3-selective antagonists have failed in clinical trials possibly due to limited bioavailability and difficulties in reaching effective concentrations at the site of action. Gefapixant, one recently developed compound which has been named after Geoff who contributed to its development, is currently undergoing clinical trials for chronic cough (Jarvis, 2020), thus confirming the key contribution of nucleotide receptors to sensory transmission. The main difficulty in translating exciting preclinical data summarized above on the purinergic modulation of glial cells to effective drugs (Figure 1) resides in the high complexity of this signaling system, on its intrinsic variability among different species, on its huge numbers of ligands, enzymes, transporters and receptor subtypes with a widespread expression which play key roles in many physiopathological conditions, thus leading to possible side effects. One recent example is represented by the demonstration that A_1_ receptors expressed by SCGs in rat DRGs reversed inflammation-induced mechanical allodynia (Xie et al., 2020). The use of agents targeting this receptor subtype would necessarily face the appearance of significant cardiovascular side effects, as long as locally-acting ligands or drug delivery strategies are applied. The use of allosteric modulators and/or partial agonists would be an additional strategy to selectively target receptors at affected sites.

Overall, the development of the first “purinergic analgesic” could be accelerated by already ongoing clinical studies for different indications. For example, selective and potent A_3_ adenosine receptor agonists are currently undergoing clinical trials for psoriasis (Phase III; Piclidenoson, CanFite BioPharma) and liver diseases (Phase II; Namodenoson, CanFite BioPharma). A phase I clinical trial with the P2X4 antagonist NC-2600 has been recently concluded by Nippon Chemiphar, showing safety and no serious side effects. Finally, novel pharmacological entities have now started to emerge which could overcome the evident difficulties in the successful delivery of “classical” chemical ligands. Monoclonal antibodies acting on human and mouse P2X4 have been synthesized showing enhanced blood-brain barrier permeability and which can be systemically delivered (Williams et al., 2019). The observed long-lasting analgesic effect in mouse models of neuropathic pain gives hope for their future clinical exploitation.

## Abbreviations

Cl-IB-MECA, 1-[2-Chloro-6-[[(3-iodophenyl)methyl]amino]-9H-purin-9-yl]-1-deoxy-N-methyl-β-D-ribofuranuronamide; DRG, dorsal root ganglia; IB-MECA, 1-Deoxy-1-[6-[[(3-iodophenyl)methyl]amino]-9H-purin-9-yl]-N-methyl-β-D-ribofuranuronamide; MRS5698, (1S,2R,3S,4R,5S)-4-[6-[[(3-Chlorophenyl)methyl]amino]-2-[2-(3,4-difluorophenyl)ethynyl]-9H-purin-9-yl]-2,3-dihydroxy-N-methylbicyclo[3.1.0]hexane-1-carboxamide; MRS5980, (1S,2R,3S,4R,5S)-4-(2-((5-Chlorothiophen-2-yl)ethynyl)-6-(methylamino)-9H-purin-9-yl)-2,3-dihydroxy-N-methylbicyclo[3.1.0]hexane-1-carboxamide.
